# Long-term nitrate removal through methane-dependent denitrification microorganisms in sequencing batch reactors fed with only nitrate and methane

**DOI:** 10.1186/s13568-018-0637-9

**Published:** 2018-06-30

**Authors:** Weiwei Li, Peili Lu, Fengguang Chai, Lilan Zhang, Xinkuan Han, Daijun Zhang

**Affiliations:** 1 State Key Laboratory of Coal Mine Disaster Dynamics and Control, Chongqing University, Chongqing, 400044 People’s Republic of China; 2 Department of Environmental Science, Chongqing University, Chongqing, 400044 People’s Republic of China

**Keywords:** Methane-dependent denitrification, Anaerobic methane-oxidation, Damo archaea, Nitrate removal, SBR

## Abstract

Denitrifying anaerobic methane oxidation (damo) bioprocesses can remove nitrate using methane as the electron donor, which gains great concern due to the current stringent discharge standard of nitrogen in wastewater treatment plants. To obtain an engineering acceptable nitrogen removal rate (NRR) and demonstrate the long-term stable ability of damo system under conditions of nitrate and methane, two sequencing batch reactors (SBRs) fed with only nitrate and methane were operated for more than 600 days at 30 °C. The NRR of 21.91 ± 0.73 mg NO_3_^−^-N L^−1^ day^−1^ was obtained which is, to the best of our knowledge, the highest rate observed in the literatures under such conditions. The temperature was found to significantly affect the system performance. Furthermore, the microbial community was analyzed by using real-time PCR technique. The results showed that the microbial consortium contained damo archaea and bacteria. These two microbes cooperated to maintain the long-term stability. And the number of damo archaea was higher than that of damo bacteria with the ratio of 1.77. By using methane as the electron donor, damo archaea reduced nitrate to nitrite coupled to methane oxidation and damo bacteria reduce the generated nitrite to nitrogen gas. The first step of nitrate to nitrite taken by damo archaea might be the limiting step of this cooperation system. SBR could be a suitable reactor configuration to enrich slow-growing microbes like damo culture. These results demonstrated the potential application of damo processes for nitrogen removal of wastewater containing low C/N ratios.

## Introduction

Denitrifying anaerobic methane-oxidizing (damo) bioprocesses has a great ecological significance and engineering potential due to the involvement of two pollutants causing eutrophication—nitrate and nitrite, and one important greenhouse gas—methane. The damo microorganisms were first enriched in a lab-scale reactor, existing as a consortium of bacteria and archaea (Raghoebarsing et al. 2006). Damo bacteria, like *Candidatus Methylomirabilis oxyfera* (*Candidatus M. oxyfera*) were demonstrated to carry out the nitrite-dependent anaerobic methane-oxidizing bioprocess (n-damo) using nitrite as the electron acceptor (Ettwig et al. 2008). These bacteria were enriched from a variety of inocula fed with methane and nitrite in different reactors (Ettwig et al. 2009; Luesken et al. 2011; Kampman et al. 2012; He et al. 2015b; Bhattacharjee et al. 2016; Ding et al. 2017b). The enrichment conditions of damo bacteria were also optimized, and the mathematical model was established (He et al. 2013; Chen et al. 2014; Hu et al. 2014; He et al. 2015a; Wu et al. 2016). “NO dismutation” is the most popular hypothesis concerning the metabolic pathway of n-damo mediated by damo bacteria (Ettwig et al. 2010; Zedelius et al. 2011; Bhattacharjee et al. 2016; Zhu et al. 2017).

In spite of the great progress made in n-damo, less attention has been paid on nitrate-dependent anaerobic methane-oxidizing bioprocess (N-damo). In a few published works on the enrichment of N-damo functional microorganism, ammonium was often used to develop anammox bacteria as the partner based on the cognition that N-damo archaea can only reduce nitrate to nitrite (Hu et al. 2011; Shi et al. 2013). From the enriched consortia of N-damo archaea, damo archaea like *Candidatus Methanoperedens nitroreducens* (*Candidatus M. nitroreducens*) was identified, and the genomics of functional microbe was investigated (Haroon et al. 2013; Arshad et al. 2015). With the detection of methyl-CoM reductase (McrA) gene and nitrate reductase in *Candidatus M. nitroreducens*, it was believed that *this microorganism* couples anaerobic methane oxidation to nitrate reduction through “reversing methanogenesis” (Haroon et al. 2013; Arshad et al. 2015). Besides, McrA gene as a functional biomarker for specific detection of *Candidatus M. nitroreducens* was founded in various environmental samples, like sediments of rivers, lakes, paddy soils (Ding et al. 2015; Lu et al. 2015; Vaksmaa et al. 2017b). Also, the engineering potential of the coupled damo and anammox system for anaerobic nitrogen removal from wastewater streams containing ammonium, nitrate and nitrite was discussed (Cai et al. 2015; Hu et al. 2015; Xie et al. 2017). The nitrogen removal rate (NRR) in a damo and anammox co-culture reached a value of 190–684 mg NO_3_^−^-N L^−1^ day^−1^, which accomplished the nitrogen removal demands of wastewater treatment plants(WWTPs) (Shi et al. 2013; Cai et al. 2015).

Considering nitrate is more ubiquitous than nitrite, it’s the main form of nitrogen oxides in many environments caused by human activities, such as agricultural runoff and effluent from wastewater treatment plants (Ding et al. 2016). Studies on damo under sole nitrate condition without nitrite might be more realistic. Vaksmaa et al. (2016) found that N-damo archaea played a more important role than n-damo bacteria in the freshwater environment via 16S rRNA qPCR technique. However, the previous understanding on N-damo process and its functional microorganism in only nitrate and methane condition are not commensurate with their importance yet. By inoculating with freshwater lake sediments and feeding with only nitrate and methane, Wang et al. (2016) and Fu et al. (2017) could not enrich damo archaea even after 13- and 6-months cultivation, respectively. These two attempts showed again that enriching damo archaea is more difficult without the existence of anammox bacteria. Vaksmaa et al. (2017a) enriched N-damo culture from paddy field soil fed with only nitrate and methane in SBR. The coupling microbial system capable of methane-dependent denitrification with only nitrate and methane provides a novel technological option for nitrogen removal from wastewater containing only nitrate and insufficient carbon source, having the advantages of less sludge production and utility of cheaper, gaseous carbon source. In China, the A-level standard of pollutant discharge in urban WWTPs (GB 18918-2002) has two types, class A and B. The discharge standard of class A is much higher than it in class B. For ammonia nitrogen, the effluent concentration in class A and B are 5 and 8 mg-N/L, respectively. For total nitrogen (TN), the effluent concentration in class A and B are 15 mg-N/L and 20 mg-N/L, respectively. The concentration of ammonia nitrogen in the effluent of WWTPs could meet the discharge demand while the concentration of nitrate could not. In that case, in the effluent of anaerobic wastewater, nitrate can be considered as the main component of TN. Detail investigations on the microbial community, process rate, long-term stability and influence factors of the methane-dependent denitrifying system under nitrate and methane conditions are necessary to further evaluate its engineering potential.

Both damo bacteria and anammox bacteria can coupled damo archaea in nitrate-dependent damo process by conducting nitrite oxidation. The products of the activity of DAMO archaea is nitrite, and a higher removal rate of nitrite product would speed up the DAMO process. The competition of nitrite between damo bacteria and anammox bacteria has been a hot topic in DAMO process. Besides, Ma et al. (2014) observed that low concentrations of ammonia (1–10 mg/L) stimulated the enrichment of *Nitrobacter winogradskyi*, a nitrite-oxidation bacterium. In order to get the maximum nitrate removal rate, anammox bacteria has been added into the reactor in the batch test to speed up the consumption of nitrite.

In this study, two SBRs fed with only nitrate and methane were operated for more than 600 days to enrich damo microorganisms from mixed sludge inoculum and its operation potential was evaluated via NRR test. The evolution of the microbial community in this system was revealed, and the effect of temperature on the process was further investigated. The objectives of this study are to evaluate the long-term performance of the N-damo in SBRs with only nitrate and methane and to obtain a high NRR for potential use for nitrate removal in wastewater with low nitrate and insufficient carbon source. These results would extend the knowledge on the N-damo process and shows a possibility that damo processes could represent an innovative technology to remove nitrate (especially at low-concentration) from anaerobic sewage effluents. In China, the lack of adequate carbon source leads the effluent of WWTPs often contains nitrate, which cannot meet the discharge demand of wastewater. By adding damo culture and flushing with methane in anaerobic wastewater, this problem might be solved.

## Materials and methods

### Inoculum and medium

Freshwater lake sediment (60 mL), paddy soil (450 mL) and methanogenic sludge (400 mL) from a local wastewater treatment plant in Chongqing, China were sampled and mixed. To remove solid impurities and organic matters, the mixture was precipitated and sieved (10 mesh), followed with cleaned (NaCl solution 0.9%) and flushed (nitrogen gas 99.99%) for three times. The pretreated mixture was then diluted with mineral medium and trace element solution to obtain a 3 L homogeneous slurry. This slurry was finally divided into two parts and inoculated into two reactors (named A and B) for the enrichment of damo microorganism. The composition of the mineral medium and trace element solutions was prepared based on the formula described by Ettwig et al. (2009). During the preparation, dissolved oxygen (DO) remained below the detection limit to keep anaerobic condition by flushing with nitrogen gas (99.99%). In the batch test coupled with anammox, anammox bacteria (100 mL, SS = 1.65 g/L, VSS = 0.77 g/L) derived from the anammox bioreactor in a previous study (Yao et al. 2015) was inoculated into reactor B.

### Operation of the reactors

Two reactors with a working volume of 2.0 L, wrapped with aluminum foil paper, were set up to run the N-damo process. And 1.5 L of the abovementioned homogeneous slurry was filled into the reactors and mixed by stirring. Temperature in the reactors were controlled using water bath circulation system. The pH value was monitored by online probes and was kept at 7.0–7.5 by manual injection of 1M HCl or 1M NaOH. The reactors were operated in the batch mode: (1) The nitrate stock solution was added into the reactors to maintain the concentration of 50–150 mg/L; (2) Methane (90% CH_4_: 5% CO_2_: 5% N_2_, vol: vol: vol) was flushed into the reactors every 2–5 days to provide substrate and keep anoxic condition; (3) Every month, about 500 mL supernatant in the reactors was exchanged with fresh medium.

In the batch test of damo coupled anammox, nitrite, nitrate and ammonium were added into reactor B at day 261. After anammox got used to the environmental condition, nitrite supply was stopped. And at the end of the batch test, ammonium nitrogen supply was stopped and 500 mL supernatant was changed to restrain the activity of anammox. Two short-term batch tests were performed when a steady state was reached. In the batch test A, nitrite (≤ 20 mg NO_2_^−^-N L^−1^ day^−1^) was added into reactor A and B. The concentrations of nitrite in the liquid and the percentages of methane in the headspace were measured 5 times/day. In the batch test B, with sufficient nutrition, nitrate and methane were sampled 5–7 times/week to calculated the consumption ratio. At the end of the batch test B, methane was replaced by nitrogen gas to observe the methane effect on conversion of nitrate.

The concentrations of nitrate, nitrite and ammonium in the liquid and the percentages of methane and nitrogen gas in the headspace were measured 2–3 times/week. The mixed liquor volatile suspended solids (MLVSS) in reactors were also tested 2–3 months/time. Based on the linear regression analysis of these measurements, the removal rate of nitrate and methane were obtained and the activities of damo culture were monitored. Reactor A and B were operated in the same condition except for the temperature conditions. The temperature in reactor B was kept at 30 °C during the whole time, while, the temperature in reactor A was adjusted from 22 to 30 °C after 144 days to explore the effect of temperature change. Operation condition details were shown in Table 1.

**Table 1:** Operational conditions of the reactors

Number	Time (days)	Substrate	T (°C)	pH
A	0–144 144–600	NO_3_^−^, CH_4_	22 30	7.0–7.5
B	0–260 261–450 451–600	NO_3_^−^, CH_4_ batch test (NO_2_^−^, NH_4_^+^, NO_3_^−^, CH_4_/NH_4_^+^, NO_3_^−^, CH_4_/NO_3_^−^, CH_4_) NO_3_^−^, CH_4_	30	7.0–7.5

### Analytical methods

The liquid samples were filtered through 0.45 μm cellulose acetate membrane filters before measurement. NO_2_^−^ was analyzed using colorimetric methods, and NO_3_^−^ was measured spectrophotometrically according to the standard test method (Federation and Association 2005). The total organic content of the liquid samples was measured by total organic carbon analyzer (TOC-L, Shimadzu, Japan). The DO value was analyzed by dissolved oxygen monitor (S4-Field Kit, Mettler Toledo, China) with the resolving power is 0.01, the accuracy is 0.2 mg/L. The MLVSS of the cultures were determined according to APHA methods (2005). Methane in the headspace was analyzed by gas chromatography (SC-3000B, ChuanYi, China) equipped with a thermal conductivity detector. The injector temperature and column temperature were set at 110 °C, and the detector temperature was set at 150 °C. Helium was used as the carrier gas at 14 mL/min. The total amount of methane of liquid and gas phases was calculated based on Henry theorem.

### Microbial structure analysis

The genomic DNA of each sample was extracted from 0.5 g sludge (dry weight) using a Soil DNA kit (OMEGA BIO-TEK, Norcross, GA, USA) according to the manufacturer’s instructions. The concentration and quality of DNA were examined using a NanoDrop 2000 spectrophotometer (Thermo Fisher Scientific, Wilmington, DE). The abundance of *M. oxyfera* bacteria and *M. nitroreducens* archaea were quantified through a real-time PCR technique with the special primer-pairs qP1F-qP1R and 345F-541R (Ettwig et al. 2009; Qian 2014). The standards were prepared using serially diluted plasmid DNA with 10^3^–10^8^ gene copies μL^−1^. Standard curves were generated by plotting the threshold cycle values vs. log10 of the gene copy numbers.

Phylogenetic analysis of *Candidatus M. oxyfer*a and *Candidatus M. nitroreducens* was conducted through analyzing 16S rRNA genes. For the *Candidatus M. oxyfera* 16S rRNA gene amplifications, nested-PCR was performed with a specific forward primer 202F (Ettwig et al. 2009) and a universal bacterial reverse primer 1492R (Dojka et al. 1998) in the first round, and a specific PCR primer-pair of qP1F-qP2R in the second round. The nested-PCR was also used to amplify the 16SrRNA gene of *M. nitroreducens* archaea, the universal primer-pair 20F-958R (DeLong 1992) was used in the first round, and the second ground was performed by the specific primer pair 142F-773R (Qian 2014). The above PCR products were respectively ligated to pMD19-T vector (TaKaRa, Dalian, China) according to the manufacturer’s instructions. Phylogenetic trees were constructed by using the neighbor joining method in MEGA 4.1 software (Tsushima et al. 2007).

### Nucleotide sequence accession numbers

The sequences reported in this study were deposited in the GenBank database under accession numbers MH094054–MH094090 (damo archaea 16S rRNA genes), MH094079–MH094109 (damo bacteria 16S rRNA genes).

## Results

### Long-term activity of the enriched culture

**Fig. 1:**
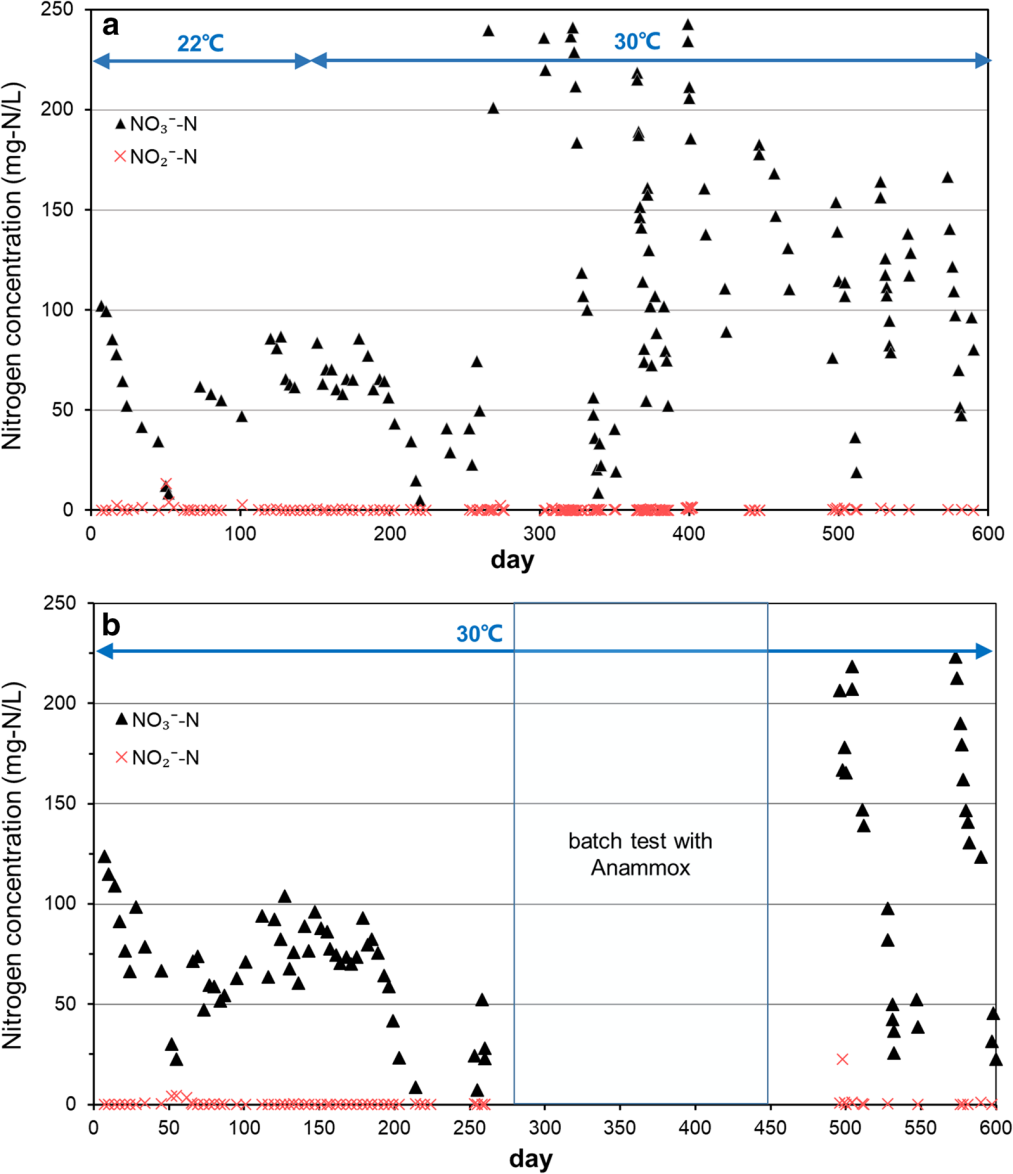
Nitrate and nitrite concentrations in the reactor during the 600 days. **a** Nitrate and nitrite concentration changes in reactor A; **b** nitrate and nitrite concentration changes in reactor B

**Fig. 2:**
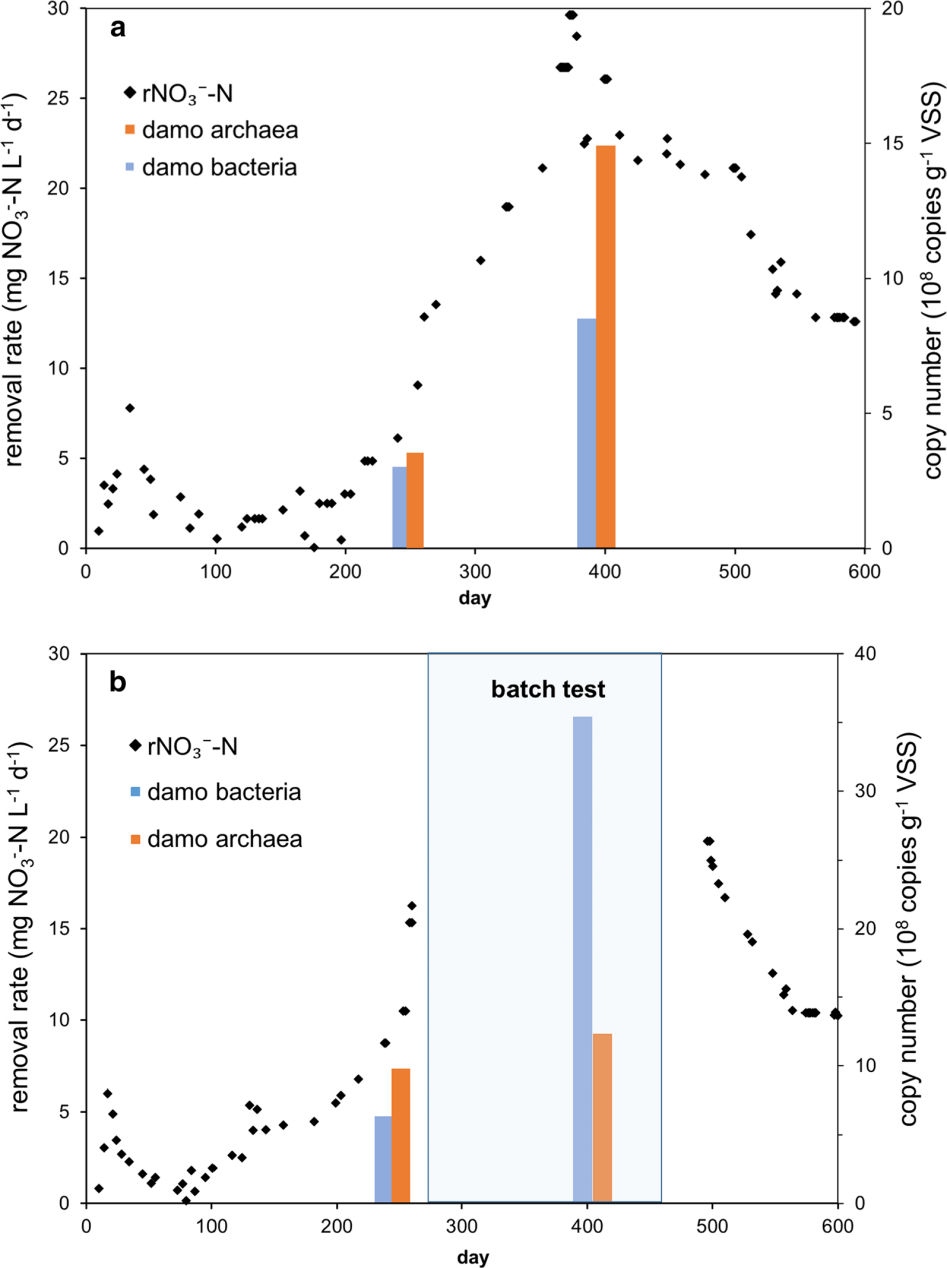
Process performance of the reactors during the 600 days. **a** Nitrate removal rates, copy numbers of damo bacteria16S rRNA gene and damo archaea 16S rRNA gene in reactor A; **b** nitrate removal rates, copy numbers of damo bacteria 16S rRNA gene and damo archaea 16S rRNA gene in reactor B

By feeding with only nitrate and methane, N-damo culture were enriched and the system were operated more than 600 days. Denitrification activity of enriched culture in SBRs was reflected by the concentration changes of nitrate and nitrite, and the results were shown in Fig. 1. The corresponding NRRs of concentration changes of nitrate were shown in Fig. 2. Reactor A went through two different temperature phases, 22 °C at 0–143 days and 30 °C at 144–600 days. During the 22 °C phase, and the NRR fluctuated in the range of 0.5–5.2 mg NO_3_^−^-N L^−1^ day^−1^ with a general trend of increase first and then descend. At day 144, the temperature in reactor A was adjusted to 30 °C. After 20 days stable in low NRRs, the NRR began to increase, followed by an acceleration stage from 4.1 to 29.7 mg NO_3_^−^-N L^−1^ day^−1^. The systems maintained at 21.91 ± 0.73 mg NO_3_^−^-N L^−1^ day^−1^ for 116 days (day 384–500). Then the NRR began to fall and kept at steady rate of 12.8 mg NO_3_^−^-N L^−1^ day^−1^ at day 575.

Expect run at 30 °C during the whole process, condition in reactor B was similar to reactor A. As shown in Fig. 2, in reactor B, the NRR fluctuated in the range of 0.7–6.0 mg NO_3_^−^-N L^−1^ day^−1^ with a general trend of increase at first and then descend from 0 to 75 days. The NRR began to increase at day 80, and the maximum NRR in reactor B reached 16.27 NO_3_^−^-N L^−1^ day^−1^ at day 260. And after day 260, a batch test with anammox bacteria was carried out to investigate the effects of anammox process on this N-damo system. In the batch test, ammonium was added into the reactor and then anammox bacteria was inoculated in the period of day 261 to day 450. During this phase, the NRR reached 66.43 mg NO_3_^−^-N L^−1^ day^−1^. From day 451, the system was recovered to fed with only methane and nitrate. Without the co-existence of anammox bacteria in the reactor, the NRR decreased rapidly and then maintained at 10.4 mg NO_3_^−^-N L^−1^ day^−1^ from day 564. During the whole processes, accumulation of nitrite in the two reactors did not occur obviously (Fig. 1). Besides, the total organic carbon in the cultures was under the detection limit and could be negligible.

### Short-term batch test of nitrogen conversion

Two short-term batch tests were performed when a steady state was reached in the system. Batch test A was conducted to identify the nitrite removal capacity of damo bacteria in the system. Since no accumulation of nitrite was found in the two reactors, artificial addition of nitrite has been inserted into reactors. Fed with methane and nitrite in two reactors, the concentration of nitrite decreased gradually. A stable nitrite removal rate of 51.91 mg NO_2_^−^-N L^−1^ day^−1^ was obtained, which was nearly two times higher than that of nitrate removal. The batch test B was undertaken to investigate the nitrogen and methane turnover rate of damo culture and verify the mass balance. The observed stoichiometry of nitrate conversion versus methane consumption was 8:4.7 ± 0.7. The conversion of nitrate ceased when methane was replaced by nitrogen gas in the batch test.

### Microbial communities of the enrichment reactors

**Fig. 3:**
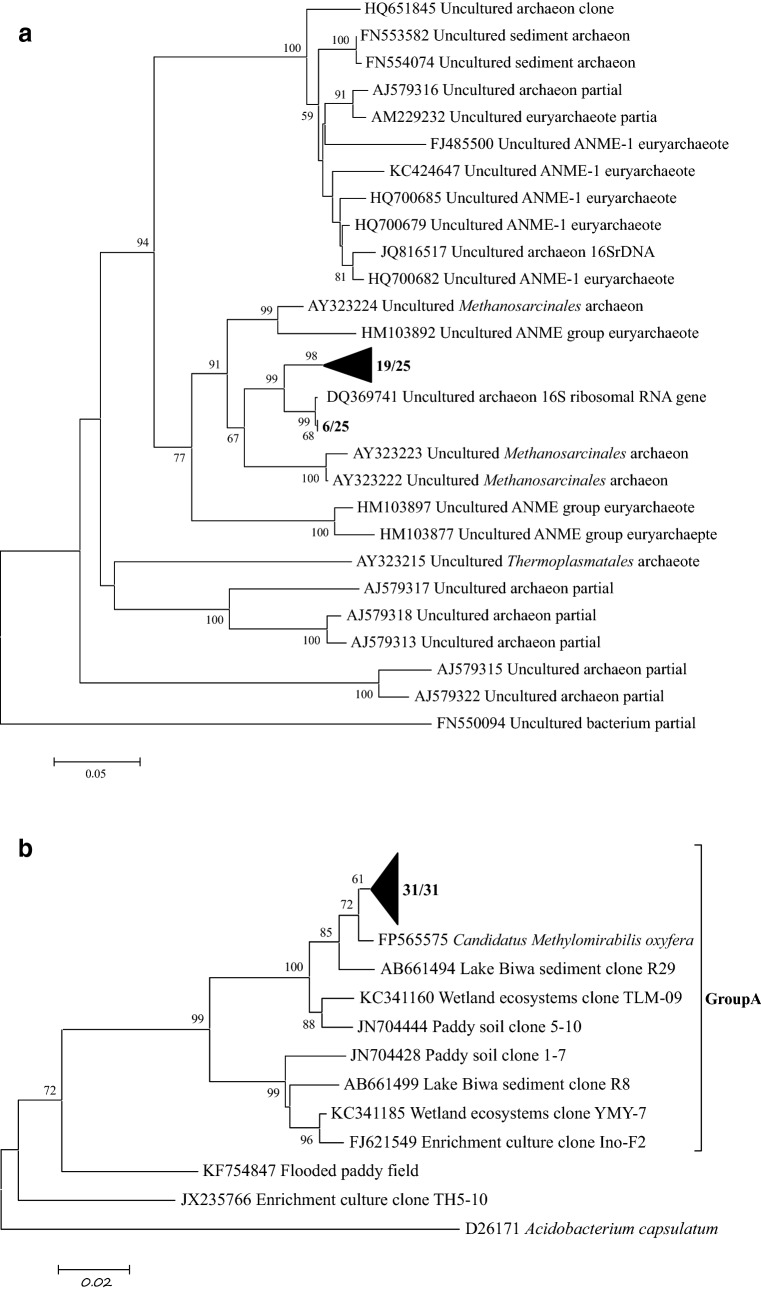
Phylogenetic analysis of the N-damo archaea and N-damo bacteria in the system. **a** N-damo archaea identified from the reactor; **b** N-damo bacteria identified from the reactor

The 16S rRNA gene of archaeal and bacterial was analyzed to determine the phylogenetic identity of the enriched cultures in the SBR reactors. A total of 25 positive clones of N-damo archaea were selected and sequenced. These sequences were related to ANME group and fell into two distinct groups of the ANME group *euryarchaeote* (Fig. 3a). And one group was found in previous ocean sediments (Quaiser et al. 2011). Sequence analysis of the bacteria showed all the sequences clustered into one subdivision which was related to *Candidatus M. oxyfera* (Fig. 3b), which was well recognized as the nitrite-dependent anaerobic methane oxidation bacteria (Ettwig et al. 2010). Similarity of the sequences between N-damo archaea retrieved in this study to *Candidatus M. nitroreducens* were 93.4–98.1% (sequences 19/25), 94.2–97% (sequences 6/25).

Real-time qPCR was conducted to quantify *Candidatus M. nitroreducens* and *Candidatus M. oxyfera* in the two reactors, and the results showed that two damo species increased with the cultivation time. In reactor A, *Candidatus M. nitroreducens* increased from 3.1 × 10^8^ to 14.7 × 10^8^ copies g^−1^ VSS from day 240 to day 400, while *Candidatus M. oxyfera* increased from 3.0 × 10^8^ to 8.3 × 10^8^ copies g^−1^ VSS. Moreover, the ratio of damo archaea to damo bacteria increased from 1.05:1 to 1.77:1. In reactor B, *Candidatus M. nitroreducens* slightly increased from 9.1 × 10^8^ to 12.1 × 10^8^ copies g^−1^ VSS while *Candidatus M. oxyfera* sharply increased from 5.8 × 10^8^ to 34.5 × 10^8^ copies g^−1^ VSS. The ratio of damo archaea to damo bacteria decreased from 1.57:1 to 0.35:1.

## Discussion

It is widely accepted that the enrichment of damo archaea is more difficult than that of damo bacteria (Hu et al. 2009, 2011; Fu et al. 2017). The physiological properties of damo archaea made them need one nitrite remover’s assist to relieve its toxicity (Hu et al. 2011). Thus, the previous studies on the N-damo often introduce anammox process by feeding ammonium (Shi et al. 2013; Ding et al. 2017b). However, there are only nitrate (no nitrite or ammonium) in some environmental media, such as underground water contaminated by nitrate or the effluent of traditional nitrification–denitrification process. Therefore, the establishment of damo culture system capable of complete nitrate removal with only nitrate and methane has significant ecological and practical meanings. In this study, N-damo microorganisms were enriched successfully in two SBRs fed with only nitrate and methane, without the assist of anammox bacteria. The nitrate removal dependent on only methane and the stoichiometric ratio of CH_4_ to NO_3_^−^ was in good agreement with the theoretical expectation of 0.63 in chemical equation (Raghoebarsing et al. 2006). And the microbial structure analysis showed that this system gained a stable population with a mixture of damo archaea and damo bacteria. The ratio of archaeal to bacterial cells in two reactors significantly increased after enrichment which was much higher value than the ratio obtained in other studies (Raghoebarsing et al. 2006; Ding et al. 2014, 2017a). No nitrite accumulation was observed during more than 600 days. All these results demonstrated that the cooperation of damo archaea with bacteria under conditions of nitrate and methane could be achieved. By using methane as the electron donor, damo archaea reduces nitrate to nitrite and damo bacteria further reduces nitrite to nitrogen gas.

The nitrite removal rate of the established coupling system of damo archaea and bacteria was nearly two times higher than that of nitrate. This result illuminated that the first step of nitrate to nitrite process conduct by damo archaea is the limiting step of the coupling microbial system. Besides, the methane affinity constant for *Candidatus M. oxyfera* has been calibrated to be 1.47 mg CH_4_ L^−1^ (He et al. 2013), which is more than one magnitude lower than that of archaeal AOM processes. Therefore, damo archaea might be critical to establish this system. After 600 days operation, the average NRR of 21.91 ± 0.73 mg NO_3_^−^-N L^−1^ day^−1^ and the maximum NRR of 29.7 mg NO_3_^−^-N L^−1^ day^−1^ were higher than previously reported in similar condition (Hu et al. 2011). The NRR of 21.91 ± 0.73 mg NO_3_^−^-N L^−1^ day^−1^ obtained in this study was almost the reported the maximum value in SBR. These rates approached the required for nitrate removal in a sidestream of WWTPs (5.6–135 mg-N L^−1^ day^−1^) or some mainstream with low nitrate concentration (Cai et al. 2015; Wang et al. 2017). Recently, Chinese government has settled a rigorous discharge standard of nitrate concentration of effluent of WWTPs. To meet the discharge standard, about extra 5 mg/L nitrate in denitrifying effluent needs to be removed. According to the NRR and HRT (3.5 month) obtained in this study, it is possible to remove nitrate in several hours, implying the potential engineering application. Additionally, methane could be obtained on-site in WWTPs and the extra methane flushed into denitrifying effluent can be removed easily. Thus, methane-dependent nitrate removal might be a cost-efficient and environment friendly technology for such low-concentrate nitrate wastewater treatment.

The reason why no damo archaea was enrichment are not clear (Wang et al. 2016; Fu et al. 2017). Using solo inoculum from freshwater sediment might be the possible reason according to the fact that no damo archaea were found in the freshwater sediment (Zhu et al. 2013). In this study, both reactor A and B went through a process conducted by heterotrophic microorganisms. During this process, heterotrophic denitrifying microorganism used residual organic matter and the NRR increased quickly. After organic matter was depleted, damo culture became the functional microorganisms. And the starting time of activity of N-damo culture in reactor B (day 75) was much earlier than that of reactor A (day 100) due to the higher temperature in reactor B. It could be concluded that the temperature may also affect the damo culture enrichment. Besides, 30 °C might be more suitable than 22 °C for the enrichment of N-damo archaea according to the variation of NRR, which has been reported in other studies (Ettwig et al. 2009; Hu et al. 2009).

A higher abundance of damo archaea in enriched N-damo system provides advantage for the study of damo archaea. In this study, the ratio of damo archaea to damo bacteria was 1.77:1, which needs to be further promoted. Recently, several new techniques were used to enrich and purify damo archaea. Ding et al. (2017a) developed a microbial fuel cell (MFC) system to purify damo archaea from enriched consortium of damo archaea, damo bacteria and anammox bacteria. After 45 days, the ratio of damo archaea to damo bacteria in the consortium increased from 1:2.7 to 2:1, which is similar to our result (1.77:1). Furthermore, combining other methods like integrated cell sorting to separate damo archaea from damo bacteria might facilitate damo archaea enrichment (Qi et al. 2017).

Until now, damo archaea were almost always enriched together with anammox bacteria by feeding with ammonium to prevent possible nitrite accumulation. The reported high NRRs were also obtained in the coupled system of damo and anammox. In this study, no nitrite accumulation was observed during the whole process in the two reactors (except the batch test in reactor B), which indicated that the system of damo archaea coupled with damo bacteria could go steadily. In the batch test of anammox addition in reactor B, the NRR reached at 66.43 mg NO_3_^—^N L^−1^ day^−1^. And after removing the cooperation of anammox (stop ammonia nitrogen supply) from the system, the NRR in reactor B dropped rapidly, and the damo culture spent more than 2 months to reach a steady status. The results showed that the NRR obtained in such a single damo system could be accelerated by the coexistence of anammox (Cai et al. 2015).

In term of reactor type, hollow fiber membrane bioreactor was reported to enrich damo archaea and obtain high NRR (Cai et al. 2015; Fu et al. 2017). However, Strous et al. (1998) demonstrated that SBR is a powerful tool to enrich slowly growing microorganisms with the advantages of efficient biomass retention, a homogeneous distribution of substrates, a long time reliable operation. Meanwhile, this study supported that SBR can be used to enrich and run damo archaea and bacteria cooperative system with only methane and nitrate. Furthermore, the starting time of N-damo system in SBRs (reflected by the increase of NRR or the increase of the number of 16S rRNA gene copies of *Candidatus M. nitroreducens*) in this study was about 75–100 days, which was shorter than those in up-flow continuous reactor of 200 days (Hatamoto et al. 2014). Vaksmaa et al. (2017a) also reported that the starting time of N-damo activity in SBR was less than 200 days.

In summary, feeding with nitrate and methane in SBRs, a microbial consortium of damo archaea and bacteria were successfully enriched. It maintained long-term stability by the cooperation mode that damo archaea reduce nitrate to nitrite coupled to anaerobic methane oxidation and damo bacteria reduce the generated nitrite to nitrogen gas using methane as the electron donor. The NRR obtained in this study is the highest in above-mentioned conditions. The competition for methane between damo archaea and damo bacteria, and the first step of nitrate to nitrite taken by damo archaea might be limiting steps of the cooperation system. N-damo showed a potential capacity in engineering applications.

## Abbreviations

Damo: denitrifying anaerobic methane oxidation
DO: dissolved oxygen
HRT: hydraulic residence time
McrA: methyl-CoM reductase
MLVSS: mixed liquor volatile suspended solids
NRR: nitrogen removal rate
SBR: sequencing batch reactor
TN: total nitrogen
WWTPs: wastewater treatment plants
